# Measure for degree heterogeneity in complex networks and its application to recurrence network analysis

**DOI:** 10.1098/rsos.160757

**Published:** 2017-01-11

**Authors:** Rinku Jacob, K. P. Harikrishnan, R. Misra, G. Ambika

**Affiliations:** 1 Department of Physics, The Cochin College, Cochin 682 002, India; 2 Inter University Centre for Astronomy and Astrophysics, Pune 411 007, India; 3 Indian Institute of Science Education and Research, Pune 411 008, India

**Keywords:** complex networks, heterogeneity measure, recurrence network analysis

## Abstract

We propose a novel measure of degree heterogeneity, for unweighted and undirected complex networks, which requires only the degree distribution of the network for its computation. We show that the proposed measure can be applied to all types of network topology with ease and increases with the diversity of node degrees in the network. The measure is applied to compute the heterogeneity of synthetic (both random and scale free (SF)) and real-world networks with its value normalized in the interval [0,1]. To define the measure, we introduce a limiting network whose heterogeneity can be expressed analytically with the value tending to 1 as the size of the network *N* tends to infinity. We numerically study the variation of heterogeneity for random graphs (as a function of *p* and *N*) and for SF networks with *γ* and *N* as variables. Finally, as a specific application, we show that the proposed measure can be used to compare the heterogeneity of recurrence networks constructed from the time series of several low-dimensional chaotic attractors, thereby providing a single index to compare the structural complexity of chaotic attractors.

## 1. Introduction

A network is an abstract entity consisting of a certain number of nodes connected by links or edges. The number of nodes that can be reached from a reference node ı in one step is called its degree denoted by $k_{i}$. If an equal number of nodes can be reached in one step from all the nodes, the network is said to be regular or homogeneous. A regular lattice where nodes are associated with fixed locations in space and each node connected to an equal number of nearest neighbours, is an example of a regular network. However, in the general context of complex networks, it is defined in an abstract space with a set of nodes N={1,2,3…N} and a set of links connecting these nodes. As the spectrum of *k*-values of the nodes increases, the network becomes more and more irregular and complex. Over the last two decades, the study of such complex networks has developed into a major field of inter-disciplinary research spanning across mathematics, physics, biology and social sciences [1–3].

Many real-world structures [4] and interactions [2, 5] can be modelled using the underlying principles of complex networks and analysed using the associated network measures [6]. In such contexts, the corresponding complex network can be weighted [7] or unweighted and directed [8] or undirected depending on the system or interaction it represents. In this paper, we restrict ourselves to unweighted and undirected networks and the possible extensions for weighted and directed networks are discussed in the end. The topology or structure of a complex network is determined by the manner in which the nodes are connected in the network. For example, in the case of the classical random graphs (RGs) of Erdös and Rényi (E-R) [9], two nodes are connected with a constant and random probability *p*. By contrast, many real-world networks are found to have a tree structure with the network being a combination of a small number of *hubs* on to which a large number of individual nodes are connected [10]. An important measure that distinguishes between different topologies of complex networks is the degree distribution P(k) that determines how many nodes in the network have a given degree *k*. For the RGs, P(k) is a Poisson distribution around the average degree ⟨k⟩ [6], while many real-world networks follow a fat-tailed power-law distribution given by $P(k)\propto k^{-\gamma }$, with the value of *γ* typically between 1 and 3 [11]. Such networks are called *scale free* (SF) [12, 13] due to the inherent scale invariance of the distribution.

Though topology is an important aspect of a complex network, that alone is not sufficient to characterize and compare the interactions that are so vast and diverse. A number of other statistical measures have been developed for this purpose, each of them being useful in different contexts. Two such commonly used quantifiers are the clustering coefficient (CC) and the characteristic path length (CPL). There are also characteristic properties of local structure used to compare the complexity of networks in particular cases, such as the hierarchy or community structure [3] in social networks and motifs [14] and superfamily profiles [5] in genetic and neuronal networks. However, a single index that can quantify the diversity of connections between nodes in networks even with different topologies, is the heterogeneity measure [15]. It is also indicative, in many cases, of how stable and robust [16] a network is with respect to perturbations from various external parameters. An important example is the network of the North American power grid [4]. Recent studies have also revealed the significance of the heterogeneity measure in various other contexts, such as, epidemic spreading [17], traffic dynamics in networks [18] and network synchronization [19].

The network heterogeneity has been defined in various ways in the literature which we will discuss in detail in the next section, where we will also present the motivations and need for a new measure. While all the existing measures are based on the degree correlations $k_{i}$ and $k_{j}$ of nodes ı and ȷ in the network, the measure proposed in this paper uses only the degree distribution P(k) to compute the heterogeneity of the network. However, we show that this new measure varies directly with the *k*-spectrum, or the spectrum of *k*-values in the network, and hence gives a true representation of the diversity of node degrees present in the network. In other words, it serves as a single index to quantify the node diversity in the network.

In this study, we also include a class of networks not considered so far in the context of heterogeneity measure in any of the previous works. These are complex networks constructed from the time series of chaotic dynamical systems, called recurrence networks (RNs) [20]. They have a wide range of practical applications [21, 22] and the measures from these networks are used to characterize strange attractors in state space, typical of chaotic dynamical systems, as discussed in §5. The diversity of node degrees in the RNs was actually one of the motivations for us to search for a heterogeneity measure that could be used to compare the structural complexities of different chaotic attractors through the construction of RNs.

Our paper is organized as follows: in the next section, we discuss briefly all the previous measures of heterogeneity and give reasons why we have to look for a new measure. The measure that we propose is based on the idea of what we consider as a completely heterogeneous network of *N* nodes, which is illustrated in §3. The proposed measure of heterogeneity is presented in §4, while §§5 and 6 are devoted to computation of this new measure for various synthetic as well as real-world networks. Our conclusion is summarized in §7.

## 2. Existing measures of heterogeneity

If we carefully analyse the heterogeneity measures proposed in the literature, it becomes clear that two different aspects of a complex network can be quantified through a heterogeneity measure. They are the diversity in node degrees and the diversity in the structure of the network. For example, the initial attempts to measure the heterogeneity try to capture the diversity in the node degrees of the network and were mainly motivated by the RG theory. The first person to propose a measure of heterogeneity was Snijders [23] in the context of social networks and it was modified by Bell [24] as the variance of node degrees:

2.1 $$\mathrm{VAR}=\frac{1}{N}\sum_{i}^{N}{\left(k_{i}-\langle k\rangle \right)}^{2},$$

where ⟨k⟩ represents the average degree in the network. Though this is still one of the popular measures of heterogeneity, its applicability is mainly limited to RGs where one can effectively define an average *k*. Another measure was proposed by Albertson [25] as:

2.2 $$A=\sum_{i,j}|k_{i}-k_{j}|,$$

which is a sum of the local differences in the node degrees in the network. This index is also not completely adequate in quantifying correctly the heterogeneity of networks with different topologies. Apart from the above two measures defined in the context of social networks, another measure [26] has recently been proposed to quantify the degree heterogeneity. It uses a measure of inequality of a distribution, called the Gini coefficient [27], which is widely used in economics to describe the inequality of wealth. Here, a heterogeneity curve is generated using the ratio of cumulative percentage of the total degree of nodes to the cumulative percentage of the number of nodes. The heterogeneity index is then measured as the degree inequality in a network. Though the authors compute heterogeneity of several standard exponential and power-law networks, the measure turns out to be very complicated and works mainly for networks of large size with N→∞. In short, none of these measures, though useful in particular contexts, truly reflects heterogeneity as represented by the diversity of node degrees in a network. A comparative study of the above heterogeneity measures has been done by Badham [28].

The second aspect of heterogeneity discussed in the literature is the topological or structural heterogeneity possible in a complex network which is especially important in real-world networks. An example for this is the measure proposed by Estrada [15] recently, given by

2.3 $$\rho =\sum_{i,j}{\left(\frac{1}{{\sqrt{k}}_{i}}-\frac{1}{{\sqrt{k}}_{j}}\right)}^{2},$$

which can also be normalized to get a measure $\rho_{n}$ within the unit interval [0,1] as

2.4 $$\rho_{n}=\frac{\rho }{N-2\sqrt{\left(N-1\right)}}.$$

If we analyse this measure closely, we find that it is basically different with respect to the earlier measures. The reason is that the measure proposed by Estrada is based on the Randic index [29] given by

2.5 $$R_{-1/2}=\sum_{i,j}{\left(k_{i}k_{j}\right)}^{-1/2}.$$

Now, the Randic index was originally proposed [30] as a topological index under the name *branching index* to measure the branching of carbon atom skeletons of saturated hydrocarbons. This index is so designed to get extremum value for the ‘star’ structure which is the most heterogeneous branching structure and is bounded by values given by

2.6 $$\sqrt{N-1}\leq R_{-1/2}\leq \frac{N}{2},$$

with the limiting values for the star structure and a regular lattice. To be specific, Estrada defines heterogeneity through an irregularity index for each pair of nodes $I_{ij}$, where $I_{ij}=0$ if $k_{i}=k_{j}$ and $I_{ij}\to 1$ for $k_{i}=1$ and $k_{j}\to \infty$. It is obvious that a measure based on this definition will be maximum for a ‘star network’ of *N* nodes compared to all other networks since there are (N−1) connections with $I_{ij}$ having maximum value.

The above discussion makes it clear that Estrada's measure elegantly captures the structural aspect of heterogeneity associated with a complex network. This is also evident in the results given by the author. Out of all possible branching structures, the heterogeneity is maximum for the star structure. While the star network has $\rho_{n}=1$, the values for networks with other topologies are much less with a typical SF network having $\rho_{n}∼0.1$. This measure is important in the context of real-world networks with different topology and structure and can be used to classify such networks, as shown by Estrada.

Our focus here is the heterogeneity associated with the diversity in node degrees (analogous to the earlier attempts of heterogeneity) to propose a measure applicable to networks of all topologies. An important difference is that we use the frequencies of the node degrees, rather than $k_{i}$ directly, to define this measure. We show that, as the spectrum of *k*-values in the network increases, the value of the measure also increases correspondingly. We call the measure proposed here *degree heterogeneity* in order to distinguish it from the measure in [15]. Also, the two measures capture complementary features of heterogeneity in a complex network. A network having high heterogeneity in one measure may not be so in the other measure and vice versa. For example, the star network is nearly homogeneous in our definition of heterogeneity, as shown below. It is also possible to correlate the robustness or stability of a network with the measure proposed here, with the SF networks having comparatively high value of heterogeneity. On the other hand, the star network is most vulnerable since disruption of just one node can destroy the entire network. To define the new measure, we require a network with a limiting value of heterogeneity to play a role similar to that of star network in the earlier measure. This network is presented in the next section.

It should be noted that some of the previous measures of heterogeneity, such as that based on the Gini coefficient [26], can give analytical expressions for networks of specific topology. Gini coefficient, being a measure in economics characterizing wealth distribution, intends to have maximum heterogeneity corresponding to a state where wealth is accumulated at one node and none is available at all other nodes indicating a star topology. However, as we have stressed in the text, the present measure is motivated by our studies on RNs from chaotic attractors. Hence we consider heterogeneity from a physical point of view as a state where wealth accumulation at each and every node is different from others. As we show later, this measure is equally effective in quantifying heterogeneity of other networks like E-R and SF and gives the correct trend as the size of the network approaches infinity.

Finally, the heterogeneity measure that we define below can be shown to have direct correspondence with the entropy measure of a complex network [31], characterized by the standard Shannon's measure of information *S*. In particular, this measure can be so adjusted to get the value zero for completely homogeneous networks and the value S→1 for the completely heterogeneous case as defined by us in this work. Though there are attempts to represent heterogeneity through entropy [32], we prefer to view the two measures as two separate aspects of a complex network. Entropy is usually associated with the rate at which a process or system (evolving) generates information. In the case of a network, each node can be considered as an information hub and links as channels for dissipating information. For the completely homogeneous network, no new information is generated while it tends to be maximum when the diversity in the node degrees is maximum. In this sense, entropy and heterogeneity are closely related and both have values normalized in the interval [0,1]. However, while entropy is a statistical measure related to loss of information in a network, heterogeneity that we consider here is basically a measure characterizing the diversity of connections between the nodes and not directly concerned with the information transfer. That is why the two measures have been treated separately here, though the values of both for the extreme cases can be made identical.

Moreover, the measure that we propose below has the following advantages:

(i) Only the degree distribution of the network is required to compute the heterogeneity in contrast to all the previous measures proposed so far.
(ii) The specific condition that we apply for the completely heterogeneous network provides analytical values for heterogeneity in terms of network size.
(iii) Based on the proposed measure, we are able to give a structural characterization index for a chaotic attractor through the construction of a complex network, namely the RN.

## 3. Completely heterogeneous complex network

Here, we present what we consider as the logical limit of a completely heterogeneous network of *N* nodes. The reader may find that this is an ideal case. Nevertheless, it helps to place the concept of heterogeneity of a complex network in a proper perspective. Consider an unweighted and undirected complex network of *N* nodes, with all the nodes connected to the network having a degree of at least one. If all the nodes have the same degree *k*, the network is completely homogeneous with the degree distribution P(k) being a *δ*-function peaked at *k*.

**Figure 1.**
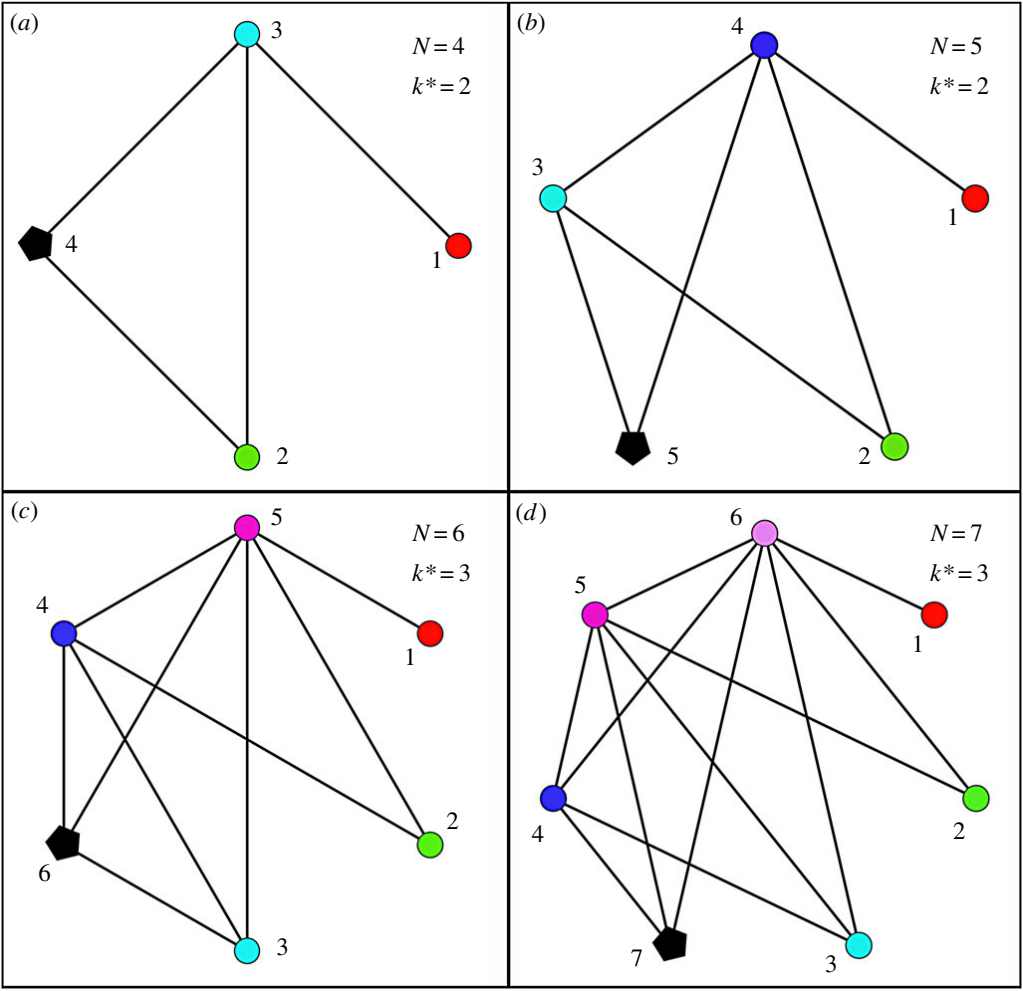
A comparison of the completely heterogeneous networks (see text) with N=4, 5, 6 and 7. In each case, all the possible *k*-values from 1 to (N−1) are present in the network as shown. One degree (one *k*-value) has to be shared by two nodes since the *N*th node will have the degree of any one of other nodes. It is empirically shown that this degree of *N*th node, denoted by $k^{*}$, is automatically fixed (if the network has all possible degrees from 1 to (N−1)) and is N/2 if *N* is even and (N−1)/2 if *N* is odd. For example, for N=4 and 5, $k^{*}=2$ and for N=6 and 7, $k^{*}=3$ and so on.

Let us now consider the other extreme where no two nodes have the same degree. The maximum possible degree for a node is (N−1). Let the nodes be arranged in the ascending order of their degree. It is obvious that the *N*th node will have to take a degree equal to that of any one of the other nodes having degree from 1 to (N−1). To find out what degree is possible for the *N*th node under the given condition, we start with taking a small number of nodes as shown in figure 1, where we show four different cases of *N* ranging from 4 to 7. In each case, the *N*th node is represented as a *pentagon shape* with its degree denoted as $k^{*}$. It is clear that if all the node degrees are to be different, there is only one possible value of $k^{*}$ for the *N*th node, which is N/2 if *N* is even and (N−1)/2 if *N* is odd.

We now give a simple argument that this result is true, in general, for any *N*. The degree of node 1 is 1 which means that it is connected only to the node with degree (N−1). That is, node 1 is not connected to the *N*th node. Node 2 is connected only to two nodes with degree (N−1) and (N−2) and hence it is also not connected to node *N*. By induction, one can easily show that the *r*th node is connected only to nodes with degree (N−1), (N−2),…,(N−r). Suppose *N* is even. When r=N/2, this node is connected to nodes with degree from (N−1) to N/2. To avoid self-loop, this node should be connected to node *N*. Thus, all nodes with higher degree from N/2 to (N−1) are connected to node *N* whose degree becomes N/2. By a similar argument, one can show that the degree of the *N*th node is (N−1)/2 if *N* is odd.

Let us now consider the degree distribution P(k) of this completely heterogeneous network. All the nodes have different *k*-values and only two nodes share the same *k*-value, $k^{*}$. One can easily show that

$$\begin{array}{rl} P(k) & =P_{0}=\frac{1}{N},\;(k\neq k^{*})\\ \mathrm{and}\;P(k) & =\frac{2}{N},\;(k=k^{*}). \end{array}$$

Our definition of heterogeneity is derived in such a way that this network has maximum heterogeneity, which is done in the next section.

## 4. A new measure of degree heterogeneity

**Figure 2.**
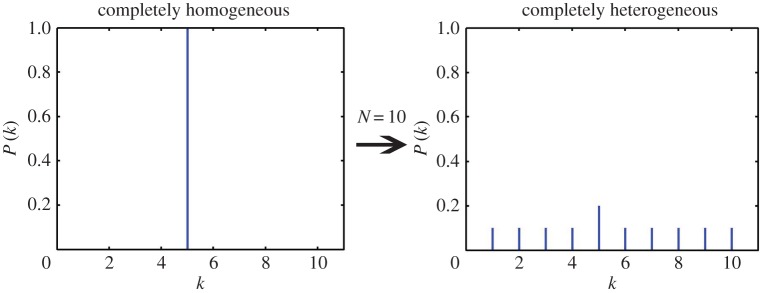
Change in the degree distribution for a typical complex network as it is transformed from complete homogeneity to complete heterogeneity, for N=10.

It is very well accepted that a network of *N* nodes with all nodes having equal degree *k* is a completely homogeneous network with P(k) being a *δ*-*function* centred at *k*. The value of *k* can be anything in the range 2≤k≤(N−1) and all these networks have heterogeneity measure zero, for any *N*. In principle, the heterogeneity of a network should measure the diversity in the node degrees with respect to a completely homogeneous network of the same number of nodes. All the measures defined so far in the literature directly use the *k*-values present in the network for computing the heterogeneity measure. Here, we argue that a much better candidate to define such a measure is P(k) rather than *k*. Since P(k) is a probability distribution, as the spectrum of *k*-values increase, the value of P(k) gets shared between more and more nodes with the condition $\sum_{k}P(k)=1$. In other words, this variation in P(k) reflects the diversity of node degrees and hence the heterogeneity of the network. A typical variation of P(k) as the network changes from complete homogeneity to complete heterogeneity is shown in figure 2. The variation of P(k) can be uniquely expressed in terms of ⟨k⟩ for an E-R network with given *p* and in terms of $k_{\min}$ for an SF network of given *γ*.

To get the heterogeneity measure, we first define a heterogeneity index *h* for a network of *N* nodes:

4.1 $$h^{2}=\frac{1}{N}\sum_{k_{\min}}^{k_{\max}}(1-P{\left(k)\right)}^{2},\;P(k)\neq 0.$$

The condition implies that the summation is only over *k*-values for which P(k)≠0. For a completely homogeneous network, P(k) is non-zero only for one value of *k*, say $k^{*}$, and $P(k^{*})=1$, making h=0, for all *N*.

We now consider the other extreme of completely heterogeneous case. From the results in the previous section, we have

4.2 $$h_{\mathrm{het}}^{2}=\frac{1}{N}\sum_{k=1}^{\left(N-1\right)}{\left(1-P(k)\right)}^{2}.$$

Putting the values of P(k) and simplifying, we get

4.3 $$h_{\mathrm{het}}^{2}=1-\frac{3}{N}+\frac{N+2}{N^{3}}.$$

This is the maximum possible heterogeneity measure for a network of *N* nodes. For large *N*, as a first approximation, we have

4.4 $$h_{\mathrm{het}}\approx \sqrt{1-\frac{3}{N}}.$$

For finite *N*, its value is <1 and as N→∞, $h_{\mathrm{het}}\to 1$. To define the heterogeneity measure $\left(H_{m}\right)$ for a network, we normalize the heterogeneity index of the network with respect to the completely heterogeneous network of same number of nodes to get the value in the unit interval [0,1]:

4.5 $$H_{m}=\frac{h}{h_{\mathrm{het}}}.$$

If *N* is sufficiently large, say N>1000 as is the case for most practical networks, $h_{\mathrm{het}}∼1$ and $H_{m}\approx h$.

**Figure 3.**
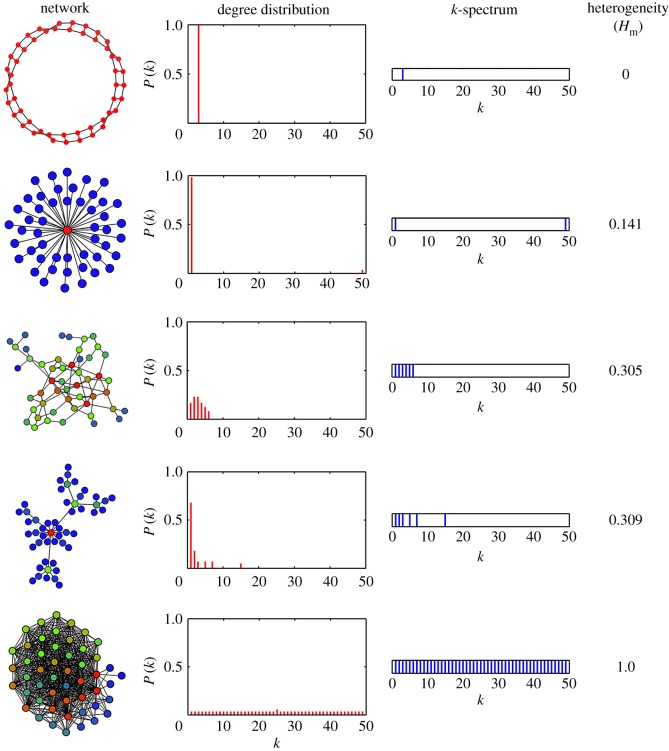
A snapshot of different types of complex networks in the increasing order of their heterogeneity ($H_{m}$), taking N=50 in all cases. From top to bottom, the nature of the network varies from completely homogeneous, star, RG, SF and finally to completely heterogeneous network. The degree distribution and *k*-spectrum are also shown for each case to indicate that $H_{m}$ is a measure of the diversity in the node degrees.

We note the following features regarding $H_{m}$:

(i) It is defined here for unweighted and undirected complex networks and represents a unique measure applicable to any network independent of the topology or degree distribution and increases with the diversity in the node degrees.
(ii) However, certain topologies have inherent limitations in diversity. For example, $H_{m}$ for a star network is very close to zero and hence the star network is nearly homogeneous in our definition. This is because, in the star topology, the degree of only one node is different from the rest of the nodes.
(iii) Since we use the counts of the node degrees rather than directly $k_{i}$ to find $H_{m}$, we cannot express the measure in terms of the elements of the Laplacian matrix, as some authors have done.
(iv) For two networks of the same size *N* independent of the topology, the measure we propose has a direct correspondence with the degree diversity in the network. To show this explicitly, we present the *k*-spectrum, the spectrum of *k*-values in the network in the form of a discrete line spectrum. In figure 3, we compare some standard networks in the increasing order of their $H_{m}$, taking N=50. For each network, we show the degree distribution (as histogram), the *k*-spectrum and the value of $H_{m}$. Note that, of different topologies, the SF network is the most heterogeneous. Here, the star network has a reasonably high value of $H_{m}$ since *N* is only 50. We also show the completely heterogeneous network with $H_{m}=1$, for comparison.
(v) The heterogeneity index *h* is defined as a measure normalized with respect to the size of the network *N*. For large *N*, since $h∼H_{m}$, the measure $H_{m}$ can also be used to compare the heterogeneities of two networks even if *N* is different. This is especially important for real-world networks where *N* varies from one network to another, as discussed in §6. However, a network with larger *N* generally tends to have lower $H_{m}$ since, to keep the same heterogeneity, the range of non-zero *k*-values should also increase correspondingly. In other words, a network with 100 nodes attains complete heterogeneity if the *k*-values range from 1 to 99, whereas, to attain complete heterogeneity for a network of 1000 nodes, the *k*-values should range from 1 to 999.

The above result also implies that for any network that is evolving or growing, for example, the SF network where the nodes are added with preferential attachment [33], the value of $H_{m}$ generally keeps on decreasing with increasing *N*. In the next section, we numerically study the variation of $H_{m}$ with different network parameters for various synthetic networks.

## 5. Degree heterogeneity of synthetic networks

In this section, we analyse three different classes of complex networks, namely, the RGs of E-R, the SF networks and the RNs derived from the time series of chaotic dynamical systems, whose details are discussed in §5.3.

### 5.1. Classical random graphs

**Figure 4.**
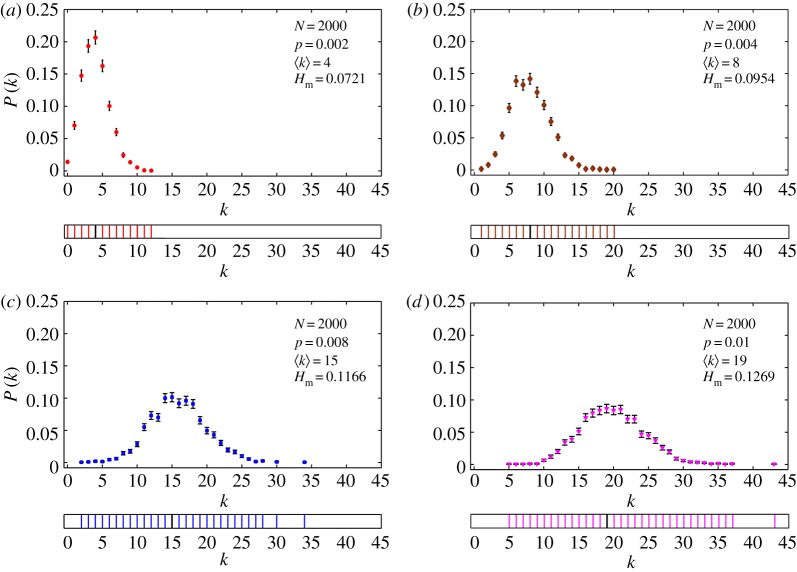
Degree distribution of E-R networks (RGs) for four different *p*-values with *N* fixed at 2000. The *k*-spectrum is shown below the degree distribution. The value of $H_{m}$ and ⟨k⟩ are also indicated in each case. Note that $H_{m}$ varies directly with the degree diversity or the spectrum of *k*-values in the network.

For RGs, the degree distribution is Poissonian centred around an average degree ⟨k⟩≡pN, where *p* is the probability that two nodes in the network is connected. In figure 4, we show the degree distribution and the *k*-spectrum for RGs of four different *p*-values with *N* fixed at 2000. The values of $H_{m}$ for all these networks are also shown. The main result here is that the value of $H_{m}$ increases correspondingly with the range of *k*-values for a fixed *N*.

We next consider how $H_{m}$ depends on *p* and *N*, the two basic parameters of the RG. The effect of changing *p* for a fixed *N* as well as changing *N* for a fixed *p* are to shift the average *k*-value of the nodes in the RG. The spectrum of *k*-values depends directly on the variance of the Poissonian profile for any given *p* and *N*. As *p* increases from zero for a fixed *N*, the spectrum of *k*-values and hence $H_{m}$ increase correspondingly (figure 4). However, as *p* increases beyond a limit for finite *N*, there are two practical problems:

(i) The fluctuations in the value of P(k) around ⟨k⟩ keeps on increasing with *p* and the profile deviates more and more from Poissonian.
(ii) The total number of edges increases rapidly with *p* and soon overshoots the computer memory making the computation practically impossible.

**Figure 5.**
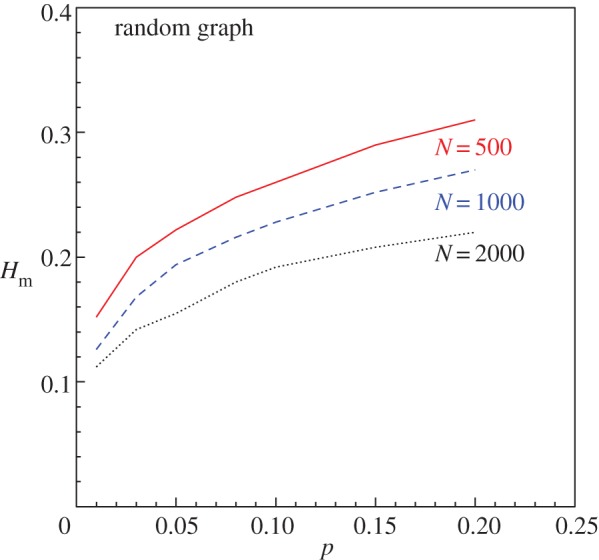
Variation of $H_{m}$ with *p* for RGs for three different fixed values of *N*.

In principle, due to the obvious symmetry of the network with respect to the transformation p→(1−p), $H_{m}$ should decrease as *p* increases beyond 0.5. As p→1, all nodes are mutually connected making the degree distribution a *δ*-function with ⟨k⟩=(N−1) and hence $H_{m}\to 0$. However, in practice, we are able to show the variation only for small values of *p* for fixed *N*, as shown in figure 5. On the other hand, by increasing *N* for any fixed *p*, one expects the profile of the degree distribution to become more Poissonian increasing the variance. But the normalization with respect to *N* decreases $H_{m}$, which is also shown in figure 5. Note that the minimum *p*-value that can be used for N=500 is 0.004 and it can be reduced for higher *N*. We have checked the variation of $H_{m}$ with *N* for smaller *p*-values, say 0.005 and 0.01, for *N* up to 5000 and have found that the decrease is approximately exponential.

### 5.2. Scale-free networks

For SF networks, the degree distribution obeys a power law $P(k)\propto k^{-\gamma }$. To construct the SF network synthetically, we use the basic scheme proposed by Barabasi *et al.* [34]. In this scheme, we start with a small number of initial nodes denoted as $m_{0}$. As a new node is connected, a fixed number of edges, say *m*, is added to the network. This number represents the minimum number of node degree, $k_{\min}$, in the network. The new edges emerging at node creation are distributed according to the preferential attachment mechanism. The two parameters, $m_{0}$ and $k_{\min}$, determines the value of *γ* as the network evolves. We have constructed SF networks of different *γ* by changing both $m_{0}$ and $k_{\min}$.

**Figure 6.**
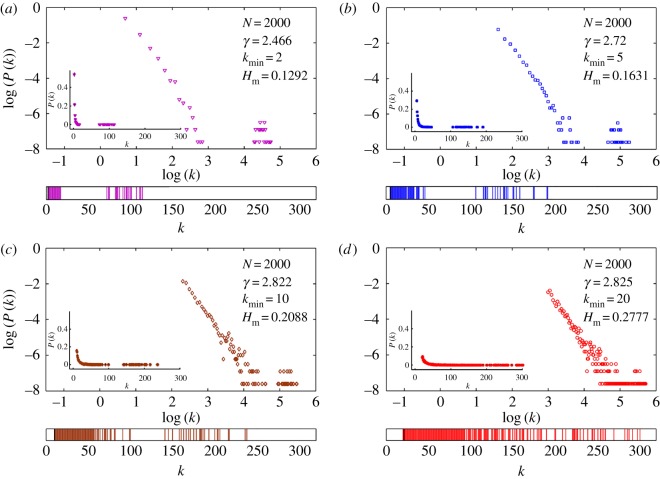
Degree distributions (inset) and the distributions in log scale along with the *k*-spectrum for synthetic SF networks with four different values of *γ* and *N* fixed at 2000. In all cases, the values of $H_{m}$ and the minimum degree $k_{\min}$ of the network are also shown. As the spectrum of *k*-values increases, $H_{m}$ increases correspondingly for a fixed *N*. Note that there appears to be a second peak with a gap in the distribution for log⁡k>4. This is a size-dependent effect due to the presence of many *k*-values having P(k) very close to zero. It is also evident from the *k*-spectrum shown below each distribution. For example, in the case (d) where the *k*-spectrum is almost continuous without a visible gap, the scaling becomes more evident.

**Figure 7.**
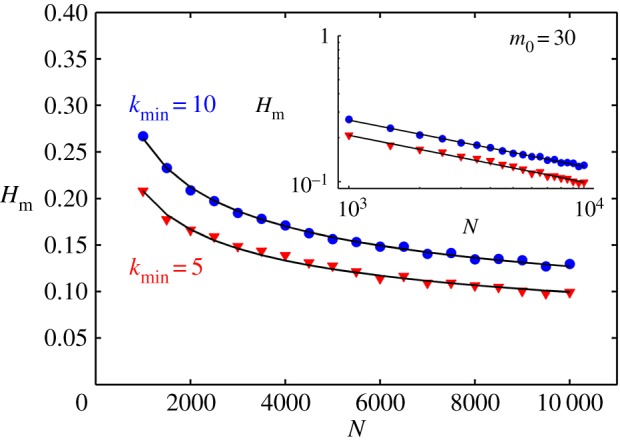
Variation of $H_{m}$ with *N* for synthetic SF networks with two different values of $k_{\min}$. The same variation is shown in the inset in log scale indicating a clear power law in both cases.

In figure 6, we show the degree distribution and the corresponding *k*-spectrum for SF networks with four different *γ* and $k_{\min}$, with *N* fixed at 2000. We find that the *k*-spectrum and hence the value of $H_{m}$ depend directly on $k_{\min}$ as can be seen from the figure. In other words, for an SF network of fixed *N*, $H_{m}$ increases as the value of $k_{\min}$ increases. The variation is approximately linear for $k_{\min}$ in the range 1–10. More interesting is the variation of $H_{m}$ with *N* for a fixed $k_{\min}$. In figure 7, we show the variation of $H_{m}$ as *N* increases from 1000 to 10 000 for two different SF networks with $k_{\min}=5$ and 10. This variation is also shown in the inset in a log scale in the same figure indicating that $H_{m}$ varies as $H_{m}\propto N^{-\rho }$, where the value of *ρ* is found to be 0.31 for $k_{\min}=5$ and 0.33 for $k_{\min}=10$ for the given range of *N*-values. However, we do not claim that this variation is, in general, a power law since we have only tested a limited range of *N*-values. This needs to be explicitly tested with other alternatives with a wider range of *N*-values.

### 5.3. Recurrence networks

Recently, a new class of complex networks has been proposed for the characterization of the structural properties of chaotic attractors, called the RNs [20, 35]. They are constructed from the time series of any one variable of a chaotic attractor. From the single scalar time series, the underlying attractor is first constructed in an embedding space of dimension *M* using the time delay embedding [36] method. Any value of *M* equal to or greater than the dimension of the attractor can be used for the construction of the attractor. The topological and the structural properties of this attractor can be studied by mapping the information inherent in the attractor to a complex network and analysing the network using various network measures.

To construct the network, an important property of the trajectory of any dynamical system is made use of, namely, the recurrence [37]. By this property, the trajectory tends to revisit any infinitesimal region of the state space of a dynamical system covered by the attractor over a certain interval of time. To convert the attractor to a complex network, one considers all the points on the embedded attractor as nodes, and two nodes ı and ȷ are considered to be connected if the distance $d_{ij}$ between the corresponding points on the attractor in the embedded space is less than or equal to a recurrence threshold *ϵ*. Selection of this parameter is crucial in getting the optimum network that represents the characteristic properties of the attractor. The resulting complex network is the RN which, by construction, is an unweighted and undirected network. The adjacency matrix A of the RN is a binary symmetric matrix with elements $A_{ij}=1$ (if nodes ı and ȷ are connected) and 0 (otherwise). More details regarding the construction of the RN can be found elsewhere [38, 39]. Here we follow the general framework recently proposed by us [39] to construct the RN from time series.

**Figure 8.**
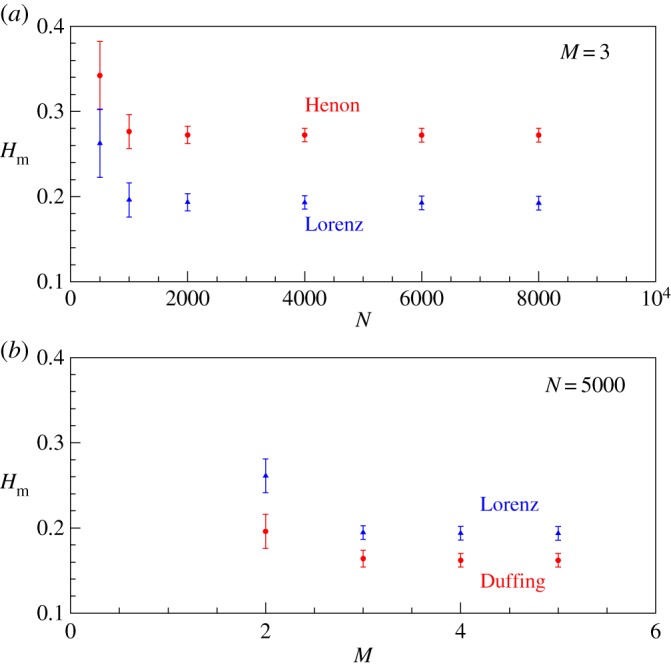
(*a*) The variation of $H_{m}$ with *N* for RNs constructed from Lorenz (triangle) and Henon (circle) attractor time series. (*b*) The variation of $H_{m}$ with *M* for fixed *N* for Lorenz (triangle) and Duffing (circle) attractors. In both graphs, the error bar comes from the standard deviation of values for $H_{m}$ computed from time series with 10 different initial conditions.

For generating the time series, we use the equations and the parameter values given in [36] for all chaotic systems. For continuous systems, we have used the sampling rate 0.05 for generating the time series. The time delay used for embedding is the first minimum of the autocorrelation. We first study how the value of $H_{m}$ varies with the number of nodes *N* for RNs. In figure 8*a*, we show the results for the Lorenz attractor and the Henon attractor. It is evident that for large value of *N*, $H_{m}$ converges to a finite value. We have checked and verified that this is true also for other low-dimensional chaotic attractors. It is found that once the basic structure of the attractor is formed, the value of $H_{m}$ remains independent for further increase in *N*. In other words, the range of *k*-values increases with *N* to keep the value of $H_{m}$ approximately constant. This result also follows from the statistical invariance of the degree distribution of the RN as has already been shown [39].

Next, we consider the variation of $H_{m}$ with embedding dimension *M*. This is also shown in figure 8*b* for two standard chaotic attractors. It is clear that the value of $H_{m}$ converges for M≥3 in both cases. This is because the fractal dimension of both these attractors are <3. We have already shown [39] that the degree distribution of the RN from any chaotic attractor converges beyond the actual dimension of the system. Thus, $H_{m}$ turns out to be a unique measure for any chaotic attractor independent of both *M* and *N*.

From the construction of RNs, the range of connection between two nodes is limited by the recurrence threshold *ϵ*. Hence the degree of a node in the RN and the probability density around the corresponding point over the attractor are directly related. For example, for the RN from a random time series with Gaussian probability distribution, every node has a degree close to the average value ⟨k⟩, because the probability density over the attractor is approximately the same. One can show that the degree distribution of the RN from a random time series is Gaussian for large *N*. Thus, the *k*-spectrum of the RN is indicative of the range in the probability density variations over the attractor, which in turn, is characteristic of the structural complexity of the attractor.

We have already shown that the measure $H_{m}$ proposed here is indicative of the diversity in the *k*-spectrum. Moreover, it is found to have a specific value for a given attractor independent of *M* and *N*. It is well known that the statistical measures derived from the RNs characterize the structural properties of the corresponding chaotic attractor. In particular, since every point on the attractor is converted to a node in the RN and the local variation in the node degree is a manifestation of the variation in the local probability density over the attractor, the measure $H_{m}$ can serve as a single index to quantify the structural complexity of a chaotic attractor through RN construction. In table 1, we compare the values of $H_{m}$ for RNs constructed from several standard chaotic attractors. In all cases, the saturated value of $H_{m}$ converged up to M=5 is shown. In each case, 10 different RNs are constructed changing the initial conditions and the average is shown with standard deviation as the error. The results indicate that among the continuous systems compared, the Lorenz attractor is structurally the most complex while in the case of two-dimensional discrete systems, the Lozi attractor is found to be the most diverse in terms of the probability density variations.

**Table 1.** Comparison of $H_{m}$ for several standard chaotic attractors.

system	Lorenz	Rössler	Duffing	Ueda	Henon	Lozi	Cat Map
$H_{m}$	0.1942±0.056	0.1684±0.042	0.1486±0.058	0.1612±0.038	0.2782±0.044	0.2924±0.072	0.1280±0.048

Finally, it will also be interesting to see how the value of $H_{m}$ is affected by adding noise to the chaotic time series. To test this, we generate data adding different percentages of noise to Lorenz data. When the value of $H_{m}$ is computed, it is found that the value reduces systematically with the increase in the noise percentage and approaches the value of noise for a noise level >50%. This result clearly indicates that the measure will be useful for analysing the real-world data.

The value of $H_{m}$ for random time series with N=2000 and M=3 is found to be 0.084±0.012. To get a comparison with the values of conventional complex networks, we compute $H_{m}$ for an RG with p=0.0035 that gives the same ⟨k⟩ as that of the RN from random time series and a typical SF network with γ=2.124 with N=2000 in both cases. The average of 10 different simulations is taken. We find that $H_{m}=0.087\pm 0.012$ for the RG, which is exactly same as the RN from random time series and $H_{m}=0.114\pm 0.06$ for the SF network.

## 6. Real-world networks and possible extension to weighted networks

**Figure 9.**
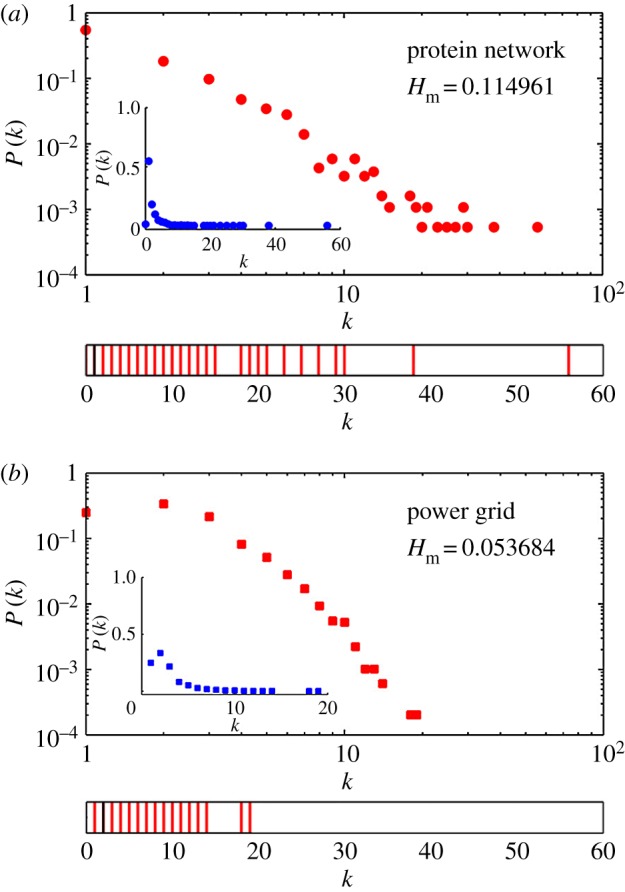
Degree distribution and the *k*-spectrum of the protein interaction network are shown in (a). In (b), the same for the network of Western Power Grid.

So far, we have been discussing the degree heterogeneity measure of synthetic networks of different topologies. In this section, we consider some unweighted and undirected complex networks from the real world and see what information regarding the degree heterogeneity of such networks can be deduced using the proposed measure. We use data on networks from a cross section of fields, such as biological, technological and social networks. In figure 9, we show the degree distribution and *k*-spectrum of two such networks. In table 2, we compile the details of these networks and the values of $H_{m}$ computed by us for each.

**Table 2.** Comparison of the degree heterogeneity measure of five real-world networks.

system	reference	*N*	$H_{m}$
US power grid	https://networkdata.ics.uci.edu [40]	4941	0.056
protein interaction	https://www.expasy.org/proteomics [41]	1846	0.1182
budding yeast	http://math.nist.gov/ [42]	2353	0.1542
US patent citation	https://snap.stanford.edu/data	7253	0.027
dolphin interaction	https://snap.stanford.edu/data [43]	62	0.386

Since we have to restrict to the case of unweighted and undirected networks, we could use only a small subset from the very large variety of real-world networks that are mostly weighted or directed. To extend the measure to directed networks, one has to consider the in- and out-degree distributions and find the heterogeneity separately. To generalize the measure to weighted networks, the distribution of the weight or strength of the nodes in the network [7, 8], rather than the simple degree distribution is to be considered and defines the measure accordingly. For example, for unweighted and undirected networks, all the links are equivalent and hence the degree of ıth node $k_{i}$ is just the sum of the links connected to node ı. On the other hand, for weighted networks, each link is associated with a weight factor $w_{ij}$ and hence the degree $k_{i}$ should be generalized to the sum of the weights of all the links attached to node ı:

6.1 $$s_{i}=\sum_{j}w_{ij}.$$

Thus, the degree distribution needs to be generalized to the *strength distribution* P(s), which is the probability that a given node has a strength equal to *s* [44, 45]. Also, unlike the degree, the strength of the node can vary continuously resulting in a heterogeneity that determines the distribution of strength among the nodes. The equation for heterogeneity can be modified accordingly for weighted networks to characterize *node strength heterogeneity*. However, it should be noted that the weight factors are assigned based on different criteria depending on the specific system or interaction the network tries to model. Modifying the measure by incorporating the specific aspects of interaction, the measure itself becomes network specific. The measure that we propose here is independent of such details and is representative of only the diversity of node degrees in a network, determined completely by the simple degree distribution.

## 7. Conclusion

Complex networks and the network-based quantifiers have become useful tools for the analysis of many real-world phenomena. Physical, biological and social interactions are increasingly being modelled and characterized through the language of complex networks. An important measure for the characterization of any complex network is its heterogeneity measured in terms of the diversity of connection reflected through its node degrees. Here, we introduce a measure to quantify this diversity which is applicable to networks of different topologies. This measure is minimum (equal to zero) for a completely homogeneous network with all $k_{i}\equiv k$. To get the upper bound for the measure, we consider the logical limit of heterogeneity possible in a network of *N* nodes where nodes of degree varying from 1 to (N−1) are present, whose heterogeneity is normalized as 1. While considering this network of limiting heterogeneity, we also prove that the degree that is repeated (or shared by two nodes) is N/2 if *N* is even and (N−1)/2 if *N* is odd.

Also, the measure that we propose here uniquely quantifies the diversity in the node degrees in the network which is characteristic of the type and range of interactions the network represents. The diversity also depends on the topology of the resulting network. For example, for RGs, the diversity is limited since most degrees are centred around the average value ⟨k⟩, while the SF networks are comparatively more diverse due to the presence of hubs. The proposed measure can quantify this diversity in the node degrees irrespective of the topology of the network.

By applying the proposed measure, we compute the heterogeneity of various unweighted and undirected networks, synthetic as well as real world. We study numerically how the heterogeneity varies for RG with respect to the two parameters *p* and *N*, while for SF networks the variation of heterogeneity with respect to *γ* as well as *N* is analysed. To illustrate the practical relevance of the measure, we analyse the RNs constructed from the time series of chaotic dynamical systems and highlight its utility as a quantifier to compare the structural complexities of different chaotic attractors. As has already been shown by us [39], the nonlinear character and chaotic dynamics underlying the time series can be distinguished from the RNs through the usual characteristic measures of complex networks like CC or CPL. However, the subtle differences between the degree distributions of RNs from different chaotic systems are not clearly evident from their CC or CPL. We find these can be quantified uniquely using the proposed measure of degree heterogeneity.

## Data accessibility

We use the open source software GEPHI http://www.gephi.org/ for the construction of all networks. All the codes used for computing the heterogeneity and other network measures are available at https://sites.google.com/site/kphk11/home.
